# Plant Physiology welcomes 15 new Assistant Features Editors

**DOI:** 10.1093/plphys/kiad631

**Published:** 2023-11-27

**Authors:** Yunde Zhao, Mike Blatt, Judy Brusslan, Mary Williams

**Affiliations:** Plant Physiology, American Society of Plant Biologists, USA; Section of Cell and Developmental Biology, University California San Diego, 9500 Gilman Drive, La Jolla, California 92093-0116, USA; Plant Physiology, American Society of Plant Biologists, USA; Laboratory of Plant Physiology and Biophysics, University of Glasgow, Glasgow G12 8QQ, UK; Plant Physiology, American Society of Plant Biologists, USA; Department of Biological Sciences, California State University Long Beach, Long Beach, California 90840, USA; Plant Physiology, American Society of Plant Biologists, USA

*Plant Physiology* is pleased to announce the new Assistant Features Editors (AFE) who are joining the editorial board in 2024 (see Fig. 1). *Plant Physiology* started the AFE program 6 years ago to help disseminate discoveries published in the journal and to train the next generation of editors and reviewers. Our AFEs are promising early-career scientists. They bring their passion for science to our journal, communicating to our readers each month some of the most exciting advances in research.

**Figure:**
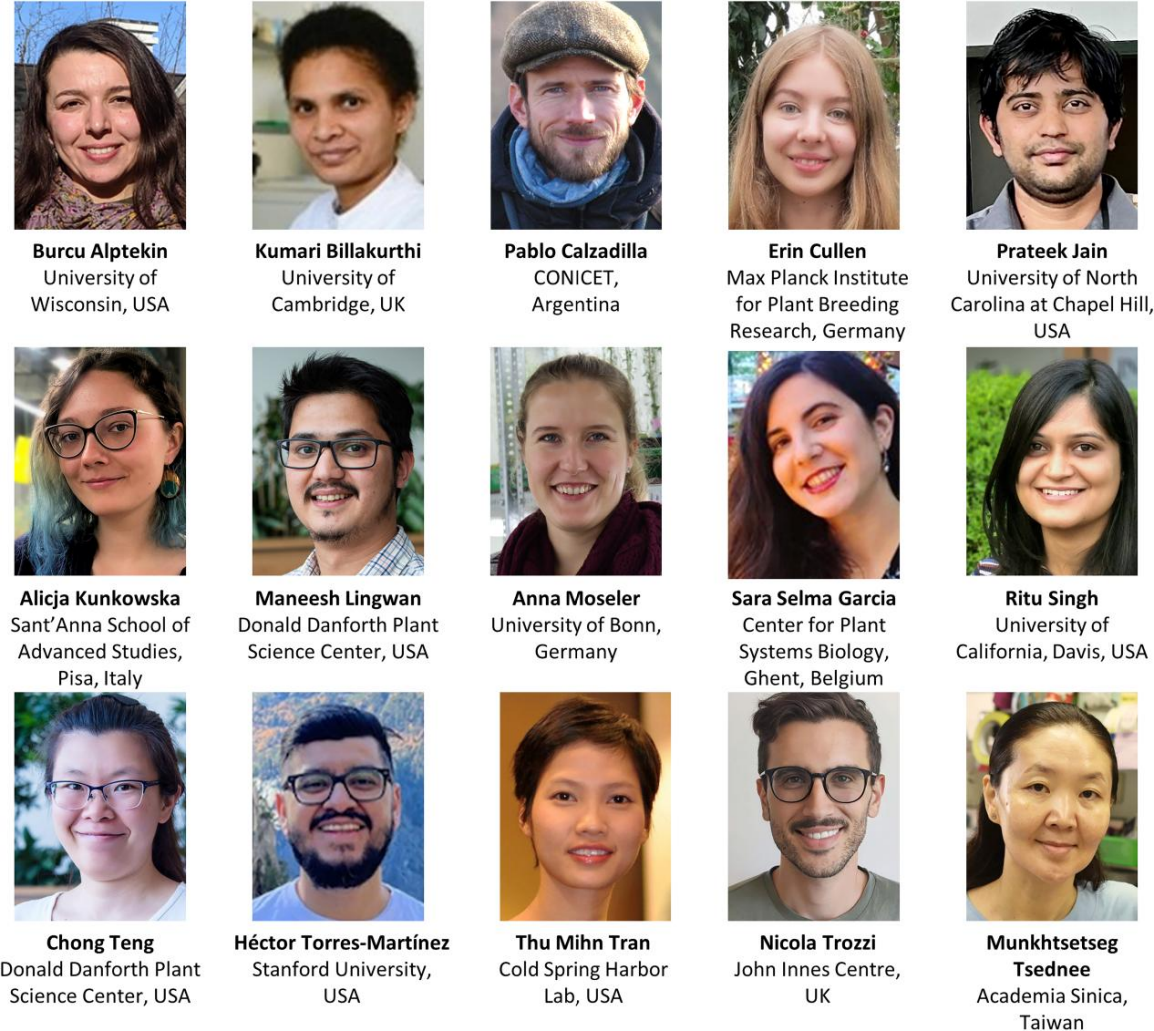


The AFEs have added substantially to the plant science community and to the journal. Their contributions have expanded our content through *News and Views* articles, blog posts, and related material highlighting content of special interest in *Plant Physiology*. They have grown professionally and will build on their experience with the journal. Many of our previous AFEs have moved onto independent academic positions after completing their terms at *Plant Physiology*. We hope to call the AFEs to serve as regular members of the editorial board when they have become more established in their careers.

The program is designed to rotate off some AFEs each year and to add new members at the beginning of each year. Thirteen of our AFEs stepped down from the editorial board at the end of 2023, and we would like to take this opportunity to thank them all for their contributions. We would also like to welcome the 15 new AFEs who started in January 2024 and will work alongside our seasoned crew (see below).

We, too, have learned much from working with the AFEs these past 6 years. We have honed the way we support them in writing *News and Views* articles and have streamlined oversight through the journal online submission system. We have invited them to write profiles of more senior members of our editorial board, which we publish on our Plantae blog. AFEs are invited to attend the annual editorial board meetings of *Plant Physiology* where they can learn the intricacies of the editorial process and contribute to discussions of *Plant Physiology* policies and operation.

So, welcome! We are thrilled to have both the new and returning AFEs with us. We want, also, to add our special thanks to the ASPB and the ASPB Publications Committee for supporting this initiative. With these new members, we are pleased to note that the expanded group reflects the broad distribution of research topics published in *Plant Physiology*.

**Welcome new Plant Physiology AFEs (2024–2025)**

- Burcu Alptekin (University of Wisconsin, USA)
- Kumari Billakurthi (University of Cambridge, UK)
- Pablo I. Calzadilla (National Council for Scientific and Technical Research of Argentina (CONICET), Argentina)
- Erin Cullen (Max Planck Institute for Plant Breeding Research, Cologne, Germany)
- Prateek Jain (University of North Carolina at Chapel Hill, USA)
- Alicja Kunkowska (Sant'Anna School of Advanced Studies, Pisa, Italy)
- Maneesh Lingwan (Donald Danforth Plant Science Center, USA)
- Anna Moseler (University of Bonn, Germany)
- Sara Selma Garcia (Center for Plant Systems Biology of VIB Ghent, Belgium)
- Ritu Singh (University of California, Davis, USA)
- Chong Teng (Donald Danforth Plant Science Center, USA)
- Héctor H. Torres-Martínez (Stanford University, USA)
- Thu Tran (Cold Spring Harbor Laboratory, USA)
- Nicola Trozzi (John Innes Centre, UK)
- Munkhtsetseg Tsednee (Agricultural Biotechnology Research Center, Academia Sinica, Taiwan)

The new AFEs are joining those who started in January of 2023, listed below, who will stay on for one more year to continue writing News and Views, help with the transition, and mentor the new AFEs.

- Dechang Cao (Institute of Botany, Chinese Academy of Sciences, Kunming, China)
- Jiawen Chen (KU Leuven, Belgium)
- Joke De Jaeger-Braet (University of Hamburg, Germany)
- Manuel Gonzalez-Fuente (Ruhr-University of Bochum, Germany)
- Henning Kirst (University of Cordoba, Spain)
- Yee-Shan Ku (Chinese University of Hong Kong, China)
- Aida Maric (University of Freiburg, Germany)
- Hannah McMillan (Duke University, USA)
- Sebastian Moreno (Sainsbury Lab Cambridge University, UK)
- Dyoni Oliveira (Center for Plant Systems Biology of VIB Ghent, Belgium)
- Lara Pereira (University of Sheffield, UK)
- Moona Rahikainen (University of Helsinki, Finland)
- Janlo Robil (Ateneo de Manila University, Philippines)
- Alaeddine Safi (Center for Plant Systems Biology of VIB Ghent, Belgium)
- Maria-Angelica Sanclemente (University of Florida, USA)
- Henryk Straube (University of Copenhagen, Denmark)
- Jiaqi Sun (Shandong University, China)
- Kyle Swentowsky (Cold Spring Harbor Lab, USA)
- Ryo Yokoyama (Max Planck Institute of Molecular Plant Physiology, Germany)

